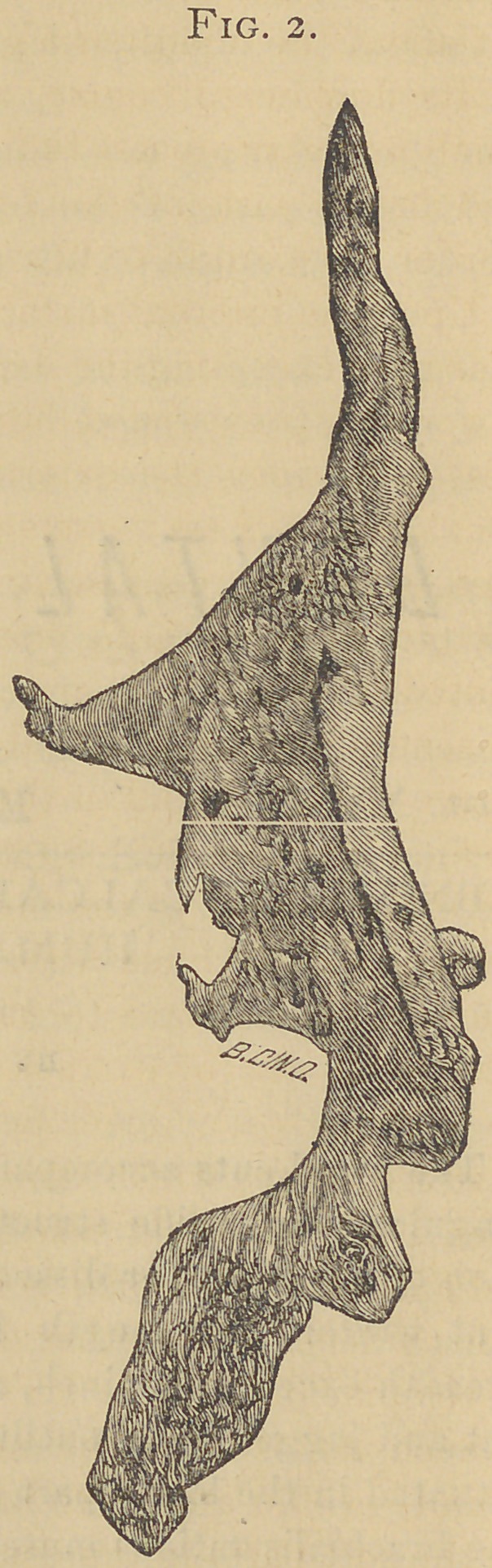# Abnormal Calcareous Formation in the Human System

**Published:** 1875-03

**Authors:** Wm. Taft


### DENTAL REGISTER.

Vol. XXIX.] MARCH, 1875. [No. 3.

### ABNORMAL CALCAREOUS FORMATION IN THE HUMAN SYSTEM.

BY WM. TAFT.

The wood cuts accompanying this article represent a very singular solid ossific structure removed from the humeral region of a subject for dissection at the dental college the present winter. Its length is over four inches, its greatest breadth exceeds one inch, and is as shown by the illustration flat and jagged in its outlines and spongy in its internal parts. Situated in the lower part of the left upper arm—imbedded in the brachialis anticus muscle—it lay obliquely across from the inner to the outer side, the downward or narrow part resting over the trochlea of the humerus and making an articulation with it by a fossa-like depression, the joint being well supplied with inter-articular cartilage and firmly held in position by strong ligaments extending laterally to the condyles. This location of the bone materially interfered with the flexion of the elbow joint, which could not be performed much beyond a right angle.

Fig. 1.

Fig. 2.

Fig. 1 represents the external surface, the upper part is broad and sends off two processes, one at each angle, the outer one being short, flat and hook shaped. The inner one longer, more rounded and broad at the base. The line from tip to tip of these processes presents an irregular curve which corresponds to the convexity of the humerus and seemed to be gradually surrounding it. Upon this surface foramina, affording passage to minute nutrient vessels, are noticeable At about the middle is a groove in which lay the ulnar artery in its downward course, and there also may be observed a small hamular process indicating the junction of the narrow and broad parts of the formation. Almost the entire inner border gave origin to fibres of the brachialis anticus muscle.

Upon the internal surface, fig. 2, tbere is nothing of particular note excepting the depression or articular surface at the lower end, previously alluded to, and the foramina for nutrient vessels as upon the external surface—it may be here stated that some of these vessels passed entirely through the specimen—the calcareous matter evidently having been deposited around them, forming canals of transmission. There is presented further in this arm an anomaly of the brachial artery, in sending off its principal branches, the ulnar and radial, just above the upper end of this bone and proceeding from thence to final distribution. So much for the description.

Of the history of the individual from whose body this formation was obtained little is known; it was a colored female whose death is said to have been caused by intemperance. The skull was of extraordinary thiokness; the other bones were also greatly developed; and it is reasonable to suppose that the causes which tended to advance normal ossification to such a degree would also manifest itself in favoring the development of abnormal bony structures or calcareous concretions whenever the other conditions were favorable for their development.

Such deposits in the muscle—in which the present was found—would appear to have been the result of some injury to the soft parts in the course of life. The consequent change in the muscular tissue would probably give it the character which the periosteum exerts in the separation of phosphate of lime from the blood and in separating the excess of acid which renders the ossific matter soluble in organic fluids. Even the injuries which the periosteum sustains and the subsequentdevelopments by the recuperative powers of life increase the power of promoting ossification. Such an increased action of the power of the periosteum is of the greatest advantage in repairing the fractures of the bones and in the formation of a callus for establishing the continuity of the solid parts. In many cases the peculiar tissue called into existence in fracture of the bones, supply the ossific material so abundantly as to endanger the occurrence of anchyloses when fractures occur in the vicinity of joints.

Of the causes which gives tissues formed after injuries the power of eliminating phosphate of lime from the blood, it seems difficult to form any satisfactory opinion. It may depend on the number of cells generated from the plastic lymph, on their peculiar size or on their power of modifying or controlling vital forces. If we follow the custom of most physiologists and reason from the doctrine of final causes, the connections of these calcareous effusions with the organic growths succeeding injuries may be ascribed to the wise provision which exists in life for maintaining the integrity of the human frame and for averting the evils which threaten the existence. The effect of this provision is not confined to the union or the repair of broken bones, for it is probable even the infiltration of calcareous matter in certain tumors may have often a beneficial influence in preventing or retarding them from assuming a malignant form.

In modern times it has been customary for writers on human physiology and pathology, to collect facts from the entire range of the animal kingdom, in order to throw light on the vital phenomena of the human frame, and on the diseases to which it is subject. Between the formation of shells in lower forms of life and the productions of bones in animals of higher organization some relation may be based, though the difference between both operations is very great. While in our bones phosphate of lime is largely in preponderance, shells consist almost entirely of carbonate of lime, they are permeated by no vessels and the crystalline form which, in most cases the calcareous matter exhibits, shows that it must be regarded as an excretion and that its deposition has little dependence on the operation of vital forces. Notwithstanding this, even shells have a provision similar to that of bones for the repair of fractures, the injured membrane and tissue supply an excessive effusion of calcareous matter, and it is said that the Chinese obtain casts in carbonate of lime by introducing metallic coins into certain kinds of shell fish (Molloscea) inhabiting their coast.

Why some should have the power of secreting calcareous matter and others devoid of the power is a question which involves much difficulty. Agassiz was the first to direct attention to the fact that the greater part of the fishes of the primitive geological ages had their vertebræ composed of unossific cartilage and that the phosphate and carbonate of lime was deposited abundantly in their scales, which from this cause are found in a good state of preservation with bright surfaces after having withstood destructive effects of an immensity of time. Even in the human frame certain causes may divert the supply of phosphate of lime which the blood affords from the bones to various localities. Ossification has been known to occur in the liver, the heart, and many of the other organs. The ossification of the arteries is common in old age and often has an important influence in bringing life to a close. Nearly every tissue in the body contains a small amount of phosphate of lime, and under peculiar derangements of the vital forces, there is an excess of it concentrated at certain localities and under rare circumstances this concentration is carried to such an extent as to lead to such formations as the one now under consideration.

These calcareous growths are also indebted for their existence to the nature of the aliment introduced into the system. It is well known that a large amount of phosphate of lime exists in milk and unbolted flour, and the use of such articles of diet may be expected to promote not only normal ossification, but also those irregular calcareous deposits which are due to some disturbance of the vital forces. Water supplied by wells and impregnated with a large amount of carbonate of lime has the same effect. The blood in its normal condition contains soluble or acid phosphate of lime, and the introduction of carbonate of lime would favor the production of the basic phosphate which is insoluble and contributes to form not only the bone but also calcareous deposits and growths of an abnormal character. Of the other possible causes which may give rise to these morbid phenomena most are involved in such obscurity that it would be inadvisable at the present state of science to enter into a discussion respecting their action.

## Figures and Tables

**Fig. 1. f1:**
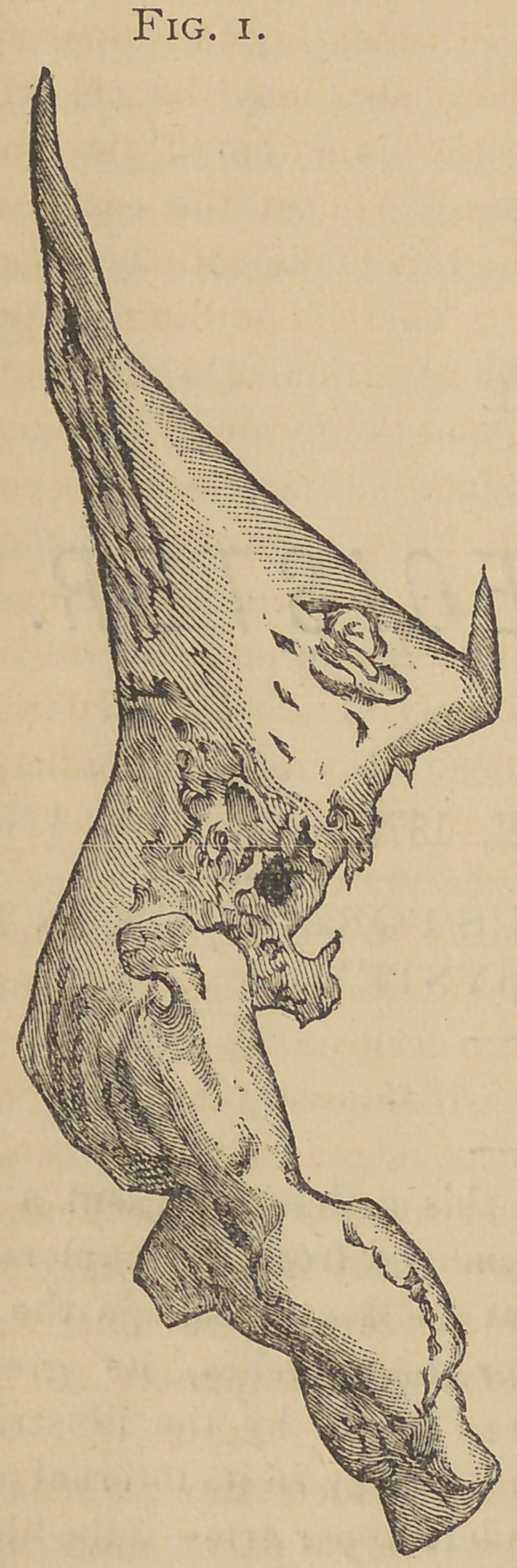


**Fig. 2. f2:**